# Community interventions to reduce child mortality in Dhanusha, Nepal: study protocol for a cluster randomized controlled trial

**DOI:** 10.1186/1745-6215-12-136

**Published:** 2011-06-03

**Authors:** Bhim P Shrestha, Bishnu Bhandari, Dharma S Manandhar, David Osrin, Anthony Costello, Naomi Saville

**Affiliations:** 1 Mother and Infant Research Activities (MIRA), GPO Box 921, Kathmandu, Nepal; 2 Centre for International Health and Development, UCL Institute of Child Health, 30 Guilford Street, London WC1N 1EH, UK

## Abstract

**Background:**

Neonatal mortality remains high in rural Nepal. Previous work suggests that local women's groups can effect significant improvement through community mobilisation. The possibility of identification and management of newborn infections by community-based workers has also arisen.

**Methods/Design:**

The objective of this trial is to evaluate the effects on newborn health of two community-based interventions involving Female Community Health Volunteers.

*MIRA Dhanusha community groups*: a participatory intervention with women's groups. *MIRA Dhanusha sepsis management*: training of community volunteers in the recognition and management of neonatal sepsis.

The study design is a cluster randomized controlled trial involving 60 village development committee clusters allocated 1:1 to two interventions in a factorial design.

*MIRA Dhanusha community groups*: Female Community Health Volunteers (FCHVs) are supported in convening monthly women's groups. Nine groups per cluster (270 in total) work through two action research cycles in which they (i) identify local issues around maternity, newborn health and nutrition, (ii) prioritise key problems, (iii) develop strategies to address them, (iv) implement the strategies, and (v) evaluate their success. Cycle 1 focuses on maternal and newborn health and cycle 2 on nutrition in pregnancy and infancy and associated postpartum care practices.

*MIRA Dhanusha sepsis management*: FCHVs are trained to care for vulnerable newborn infants. They (i) identify local births, (ii) identify low birth weight infants, (iii) identify possible newborn infection, (iv) manage the process of treatment with oral antibiotics and referral to a health facility to receive parenteral gentamicin, and (v) follow up infants and support families.

*Primary outcome*: neonatal mortality rates. *Secondary outcomes: MIRA Dhanusha community group*: stillbirth, infant and under-two mortality rates, care practices and health care seeking behaviour, maternal diet, breastfeeding and complementary feeding practices, maternal and under-2 anthropometric status. *MIRA Dhanusha sepsis management*: identification and treatment of neonatal sepsis by community health volunteers, infection-specific neonatal mortality.

**Trial Registration no:**

ISRCTN: ISRCTN87820538

## Background

### The importance of newborn survival

The fourth and fifth United Nations Millennium Development Goals are linked explicitly with maternal and child survival[1]. 11 million children under five die each year, 4 million of them in the neonatal period and 98% in developing countries [2,3]. The decline in post-neonatal mortality through immunisation, control of diarrhoeal disease and treatment of acute respiratory infection has increased the relative importance of the newborn period [3,4].

Nepal has an infant mortality rate of 51 per 1000 live births and a neonatal mortality rate of 34: [5] newborn deaths comprise 66% of child deaths. Primary and secondary care is deficient, most women have no antenatal care and most deliveries occur at home. The 2006 Nepal Demographic and Health Survey showed that 13.5% of rural women and 4.3% of the poorest wealth quintile had given birth in a health facility and that skilled attendance at delivery only reached 14.3% of rural women[5]. Most stillbirths and neonatal deaths also occur at home, and many might be avoided with changes in antenatal and newborn care practices. Efforts to improve access to care have to deal with difficult terrain and lack of infrastructure, limited expenditure on public health, suboptimal quality of care, a high turnover of service providers, a lack of drugs and supplies, and a lack of ownership of health programmes by communities.

### Community intervention to improve newborn survival

In India, the SEARCH group reported a controlled study from a rural population of about 80,000 in Gadchiroli, Maharashtra[6]. At baseline, almost half of newborn infants encountered high-risk morbidity, commonly due to sepsis. In intervention villages, SEARCH trained traditional birth attendants, introduced health education, and developed a new cadre of supervised village health workers to visit newborn infants at home, identify warning signs and manage sepsis with a combination of injectable and oral antibiotics. By the third year of the intervention, the neonatal mortality rate was 26 per thousand live births in intervention and 60 per thousand in control villages. So far, the employment and management of health workers outside the government system, and the reluctance of government and professional bodies elsewhere to sanction delivery of injectable antibiotics by community health workers, have limited the model's scalability. However, adaptations of the model have been evaluated in Bangladesh,[7] India,[8] and Pakistan, [9] using home visits by community health workers for rapid referral but without using injectable or oral antibiotics in the home, and with varying degrees of success.

### Community intervention to treat childhood infections

It has become clear that the management of childhood infections - particularly acute respiratory infections - by community health volunteers is feasible and effective. The WHO recommends a combination of three strategies: improving quality of care and accessibility at first-level public health facilities, improving quality of care in the private sector, and increasing access to care through the work of community health workers. These latter can be trained to assess, counsel, treat, refer and follow up children with pneumonia in the community[10]. Meta-analysis suggests that such approaches can lead to a reduction of 24% in total mortality and 36% in pneumonia mortality in children under five years old[11].

Much of the work on community management of pneumonia was done in Nepal. The model has been taken to scale through 54 000 female community health volunteers. At present, the programme covers infants and children over the age of two months, but there is concern that it should be expanded to cover younger infants. The Morang Innovative Neonatal Intervention (MINI), implemented by the Ministry of Health and Population, with technical support from John Snow International and funding from The Bill and Melinda Gates Foundation through Saving Newborn Lives and Save the Children USA, trains existing Female Community Health Volunteers and peripheral government health workers to identify and manage neonatal infections [12-14] using oral antibiotics in the home (cotrimoxazole) and to refer rapidly to the nearest health facility for treatment of sepsis cases with injectable gentamicin. This pilot study has shown good referral rates and reduction in case fatality rates, but there is no control group comparison, and it is uncertain whether these results could be reproduced at scale in the government system.

### Working with women's groups

The Warmi project, implemented in Bolivia in a poor, rural population of 15,000 with little health system infrastructure, worked with women's groups to encourage participatory planning for mother and infant care,[15] and documented a fall in perinatal mortality from 117 to 44 per thousand births over three years. Building on this example, we conducted the MIRA Makwanpur trial in a population of 170,000 in a middle hills rural district of Nepal. One woman facilitator per population cluster of 7000 facilitated monthly meetings with women's groups to address the issues of pregnancy, childbirth and newborn health. Each group moved through a participatory planning cycle to explore perinatal care strategies and solutions[16]. The large cluster randomised controlled trial suggested that participatory work with women's groups could reduce neonatal mortality by 30%[17]. From a conservative viewpoint, the study at least demands replication and assessment of modifications that would make it scalable and institutionally sustainable within a national public health framework. A recent replication trial conducted in India's Jharkhand and Orissa states achieved a 45% reduction in neonatal mortality in a population with very high baseline mortality rates [18].

There is no doubt that improvements to women's diets are needed, particularly from a life-cycle perspective, and little doubt that behaviour change could improve the health of both women and their infants. However, programmatic effectiveness to achieve behaviour change remains unclear[19,20]. Nutrition is intimately linked with social development, and we propose that a community mobilisation intervention that has led to improvements in maternal and neonatal survival could also be a route into dietary behaviour change. The demand-side approach is a model that may sustain a range of issues. The MIRA Makwanpur study addressed the issues of pregnancy, delivery and newborn care. It did not, however, include any discussions about diet and nutrition. There is ample scope for such discussions to be included in one or more action research cycles, and for local strategies to improve the nutritional status of women to be formulated. We should like to examine the potential for including nutritional issues in the community group work.

### Nepal's Female Community Health Volunteers

The Female Community Health Volunteer (FCHV) role is a grassroots worker. She is nominated by a mother's group, becomes its member secretary and is responsible for facilitating the group itself and for building linkages with the health system at village level. In general, one FCHV is allocated per ward, of which there are 9 in each Village Development Committee. There are up to 54 000 FCHVs in Nepal: 48 550 in rural village development committees and 5450 in municipalities.

Initial training on primary health care lasts 18 days. Mother and child health and family planning are a particular focus. FCHVs receive five days of refresher training per year, usually on issues such as HIV/AIDS, sexually transmitted infections, nutrition and the Integrated Management of Childhood Illness. Supervisory responsibility rests with the District Public Health Office, and FCHVs meet with their local health facility supervisors once per month and the District Public Health Officer every six months.

The FCHV has a number of specific responsibilities: facilitation of the mother's group, counselling on family planning and distribution of contraceptives, contribution to the community component of the integrated management of childhood illness, health education, safer motherhood referral services, immunization with an emphasis on oral polio vaccine and tetanus toxoid, distribution of vitamin A, anthelminthics and iron supplements, management of minor illnesses and injuries, and nutrition education [21]. FCHVs are not salaried, but receive support for attending training and incentives for particular activities.

The SEARCH findings from rural India have shown that treatment of newborn infections in the community using paid workers to visit homes and provide injectable antibiotics can significantly reduce neonatal mortality [6]. The intervention was complex and scaling up presents difficulties for policymakers. Nepal's Acute Respiratory Infection programme, however, has been successful in achieving broad coverage by FCHVs to provide oral antibiotics and referral advice for children with signs of acute respiratory infection. At present, the programme covers infants and children over the age of two months. We wish, therefore, to do two things: first, to build on and simplify the Indian model on a framework that targets scalability; second, to test the effectiveness of expanding the programme activities to cover the newborn period, a move supported by the Nepal Family Health Programme and others. We have collaborated with and learnt from the MINI project, which has developed extensive training and monitoring packages for community-based management of newborn infections.

## Methods/Design

### Trial objective

We are carrying out a trial of two models of intervention to improve child survival.

### MIRA Dhanusha community groups: a participatory intervention with women's groups

FCHVs are supported in convening monthly women's groups. Nine groups per cluster (270 in total) work through an action research cycle in which they (i) identify local issues around maternity, newborn health and nutrition, (ii) prioritise key problems, (iii) develop strategies to address them, (iv) implement the strategies, and (v) evaluate their success. They then move on to a second action cycle focused on nutrition, in which they follow the same steps (i to v), but concentrate on maternal and infant nutrition and associated aspects of postpartum care. Our main objective is to test the replicability and scalability of the women's group interventions focused on maternal and newborn health described above (from Makwanpur, Nepal and Jharkhand and Orissa, India) in reducing neonatal mortality and, in the second action cycle, to explore the potential for this approach to improve maternal and infant nutrition.

### MIRA Dhanusha sepsis management

FCHVs are trained to care for vulnerable newborn infants. They (i) identify local births, (ii) identify low birth weight infants, (iii) identify possible newborn infection, (iv) manage the process of treatment with oral antibiotics (Amoxicillin) and referral to a health facility to receive parenteral gentamicin, and (v) follow up infants and support families. We aim to build on operational research studies in Nepal that have shown that the approach is feasible, by testing the effect on mortality at population level.

### Design

**Figure 1:**
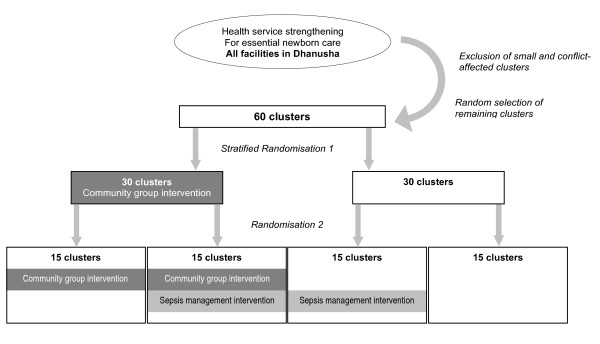
**Design of the study**. Essential Newborn Care training was provided throughout the district, followed by exclusion of small and conflict-affected clusters and stratified block randomisation on the basis of cluster size to women's groups intervention and control. A second block randomisation allocated equal numbers of clusters to sepsis management intervention within each women's group study arm.

The methodology is a cluster randomized controlled trial. A cluster design has been chosen because the unit of randomisation is the village development committee rather than the individual mother and child. The study has a factorial design, summarized in Figure 1. 60 village development committee clusters are selected randomly. Each cluster has a population of about 8000. 30 clusters are allocated randomly to receive the women's group intervention and 30 act as controls. From each of these two arms, 15 clusters are randomly allocated to receive the community health volunteer training intervention.

#### Primary Research questions

1. What is the impact of a participatory intervention with women's groups on neonatal mortality?

2. What is the impact on neonatal mortality of training community health volunteers on the recognition and management of neonatal sepsis?

#### Secondary research questions

1. What is the impact of a participatory women's group intervention that focuses on nutrition and feeding practices on the nutritional status of women and their young children?

2. What is the impact of a participatory intervention with women's groups on infant and under-2 mortality?

3. What is the impact on early infant mortality of training community health volunteers on the recognition and management of neonatal sepsis?

#### Hypotheses

1. A participatory intervention with women's groups will be associated with reductions in neonatal mortality.

2. Training of community volunteers in the recognition and management of neonatal sepsis will be associated with increases in identification and treatment of neonatal sepsis, and improvements in neonatal mortality.

3. A participatory intervention in which women's groups discuss diet and nutrition will be associated with positive changes in maternal diet and infant feeding, and in anthropometric status of women and their young children.

#### Setting

The programme is implemented in Dhanusha district, where MIRA has been working since 2001. The administrative centre in Janakpur municipality - which is excluded from the trial - manages a local team of researchers, health workers and field workers, as well as maintaining excellent relations with both hospital collaborators and the District Public Health Office. Dhanusha is a *terai *(lowland) district of 1180 km^2^. According to the 2001 census, it had a population of 670 000 (about 20 000 per doctor) and was the fifth most populous district in Nepal. The average household size was 5.72[22]. Dhanusha's human development index, a composite measure of three dimensions of human development--a long and healthy life, access to education and a decent standard of living [23] was 0.449 (rank 43/75), compared with the national average of 0.471, in 2004[24]. 89% of Dhanusha's population are rural, 42% have access to toilet facilities and 93% have access to improved sources of drinking water[22,25]. The commonest language spoken is Maithili and the majority religion Hinduism (88.7%), followed by Islam (8.7%).

#### Target group

The main beneficiaries of the study are women of reproductive age (15-49 years), of whom there were 140 000, according to Dhanusha District Public Health Office data for 2004-5, and infants under one year of age, of whom there were 19 000. 26 000 pregnancies were expected annually in the district and the crude birth rate was 29.5 per 1000 population. About 72% of women cannot read or write[26].

Key participants are women who either join community groups or have a pregnancy. The women's group intervention involves social mobilisation and group membership and activities are not restricted to women of reproductive age. Since the aim is to improve the situation of pregnant women and their newborn infants, any participant who may affect this may be involved. Particular stakeholders may be older women, male community members, health workers and local opinion formers. Experience from other sites has shown that women form the core membership of groups and that both women of reproductive age and older women get involved. All women and their newborn infants are eligible to participate in the data collection exercise, for which enrolment occurs in the postnatal period.

#### Intervention group activities: MIRA Dhanusha community groups

**Figure 2:**
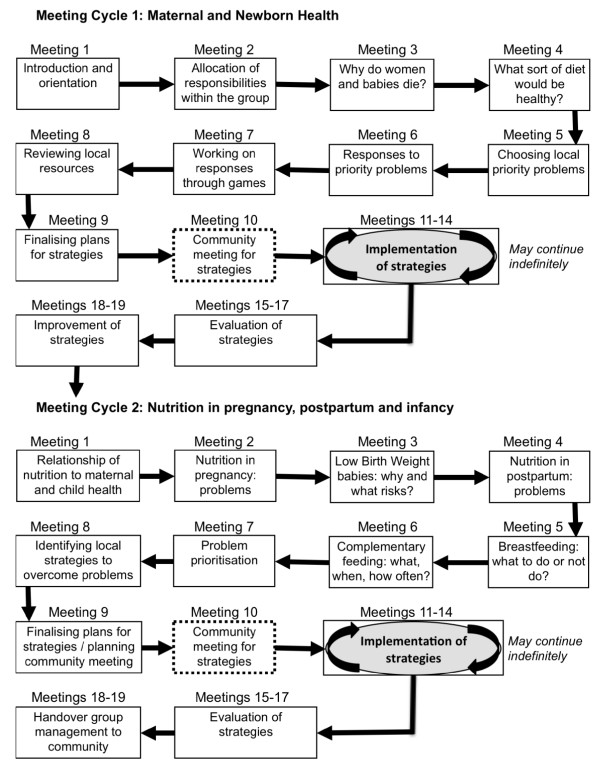
**The sequence of women's group activities**. Meetings conducted on a monthly basis between May 2007 and April 2011 by 270 groups in 30 study clusters. Strategies initiated after meeting 10 in both cycles may be implemented continually while meetings on other topics are ongoing and also after the end of the intervention period.

Each FCHV is already tasked with women's group activities under current government guidelines, although most groups were not meeting regularly before the study began. FCHVs are trained and supported in acting as facilitators for women's groups, according to guidelines developed by the intervention team. Groups are strengthened where they already exist, and initiated where they do not. In general, there is one women's group per ward, corresponding with nine per cluster and 270 over the whole intervention area. FCHVs convene women's groups monthly. Because around 60% of FCHVs in Dhanusha are illiterate, a literate co-facilitator, selected by the community group, assists the FCHV in running the group. The primary newborn and maternal health cycle, following a similar agenda to that used in the MIRA Makwanpur trial,[16] but with additional nutritional content, comprises nine pre-implementation meetings, a community meeting to share problems, plans and enlist community support, an implementation phase and an evaluation (summarized in Figure 2). The monthly meetings proceed from discussions of illness, mortality and poor nutrition in mothers and babies, via discussions of common problems in the community and the collection of local information by group members, to prioritisation of the most important problems. Following this, strategies to address these problems are formulated, shared with the wider community and implemented. Finally, the groups themselves evaluate the effects of the strategies and then move on to further activities and discussions. The FCHVs assist the women's groups in devising local strategies to realistically tackle the issues in a resource-constrained context.

Community commitment and ownership are essential to ensure implementation. The FCHV's brief is to activate and strengthen women's groups, support them in identifying and prioritising maternal and neonatal problems, help to identify possible solutions and support the planning, implementation and monitoring of the solution strategies in the community.

Formative research on beliefs and practices in pregnancy and postpartum informed the design of a second cycle of monthly meetings focused on nutrition. The meetings cover socio-cultural problems or barriers associated with the following: nutrition in pregnancy; high prevalence of low birth weight; nutrition, hygiene and postpartum care of the mother; early and exclusive breastfeeding; and complementary feeding of the infant at six months (see Figure 2 for sequence of meetings). The pattern of the second action research cycle is the same as cycle one: after a pre-implementation phase covering the topics outlined, a community meeting is held to enlist support and share ideas. Then, over several months the groups implement strategies to address the nutrition-related and/or maternal and child health related problems they prioritise, and finally evaluate these strategies. Strategies initiated during the first action cycle may continue to be implemented by the groups during the second cycle and also after the implementation period.

Process evaluation is used to document each group's progress through the monthly meeting cycles, recording group attendance, outcomes or decisions from individual meetings, problems prioritised, strategies implemented, progress with strategies and findings from the participatory evaluations.

#### Intervention group activities: MIRA Dhanusha Sepsis management

Our focus is on training FCHVs in the recognition and management of neonatal sepsis. MIRA has programmatic experience in health service strengthening at all levels. This relates mainly to the training of cadres from FCHVs to doctors in essential newborn care. MIRA is the key trainer in this field in Nepal, and has produced materials for all levels of cadre in collaboration with the National Health Training Centre of the Department of Health Services. We are building on this experience to devise and implement a new training and support programme for FCHVs. This programme focuses on the identification and management of neonatal sepsis. Its technical content was developed in consultation with national and international authorities, and is in line with multilateral maternal and child health programming.

**Figure 3:**
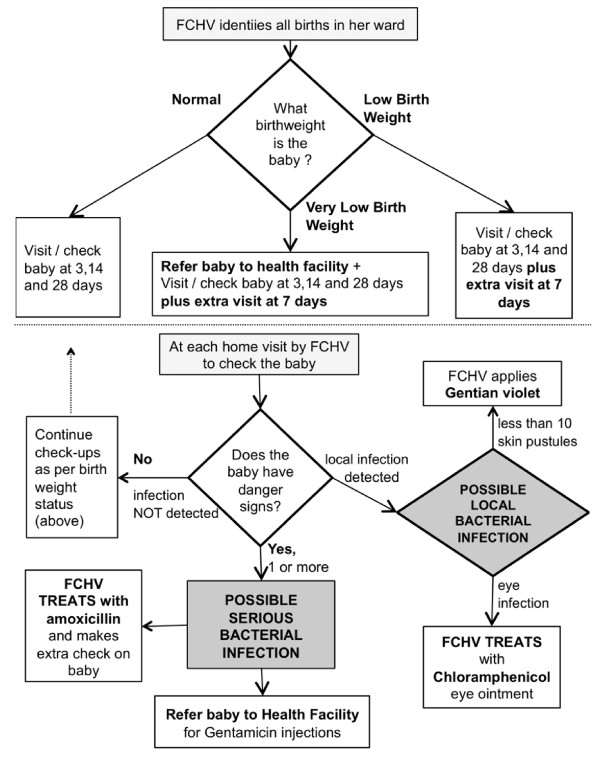
**Flowchart of community-based sepsis management of babies 0-2 months by Female Community Health Volunteers in Dhanusha**. Actions and decisions undertaken by FCHVs when checking birth weight and for signs of possible local bacterial infection or possible severe bacterial infection in babies born in their working areas.

FCHV activities are summarized in Figure 3. Her key role is to identify within 24 hours all babies born in her ward working area, and provide their families with birth record forms to facilitate registration. The FCHV routinely follows-up a baby, regardless of birth weight categorisation, at 3, 14 and 28 days. She identifies low birth weight infants with a simple colour-coded spring balance, and provides advice and support on their care. Low birth weight babies are identified by a red or yellow zone on the spring balance, and referred to a health facility if very low birth weight (in the red zone of the scale). She is also trained to identify local and severe bacterial infection in any baby by recognition of 10 danger signs, including fever > 38°C, temperature < 36°C, lethargy, not feeding, weak or absent cry, fast breathing, grunting, chest in-drawing, umbilical infection with redness all around or pus seepage, and more than 10 small skin pustules or one big abscess or pustule. Local infections are treated on site and followed up to make sure that they are settling. Skin infections are treated with gentian violet and oral amoxicillin is provided if more than 10 skin pustules or an umbilical infection are found. Eye infections are treated with chloramphenicol eye ointment. Possible severe bacterial infection, as identified by the presence of one or more danger signs, triggers a sequence of activities. The FCHV gives the first dose of oral antibiotic (amoxicillin) in the home and shows mothers and carers how to administer further doses by suspending it in breast milk and spoon-feeding it to the baby morning and evening for seven days. The FCHV then calls a MIRA supervisor to check on the baby and also arranges and checks on referral to a health facility. The FCHV checks on the baby on the third day after first administering amoxicillin. If the baby has improved, she records this and again visits on the seventh day after starting the amoxicillin. If the baby does not improve at three days the FCHV calls the MIRA supervisor to visit and check on the baby again. Although checking at days three and seven after identifying the infection is required, where possible the FCHV checks daily on babies with infection as they are usually living nearby. In the case of low and very low birth weight or possible infection in the first week, she visits for follow-up at 3, 7, 14 and 28 days (an extra visit at 7 days on top of the routine timetable).

The concept of home treatment is a new one. It has arisen from two realities: (a) that community workers are already treating infection in older infants with oral antibiotics, and (b) that referral advice is not always acted upon. There are strong cultural taboos against taking newborn infants out of the home and opposition to the concept of giving them injections. In this situation, we feel that - even though the counsel of perfection is to give the infant parenteral antibiotics - oral antibiotics represent a first step and a realistic response to the prevailing situation[27].

The choice of oral antibiotic is amoxicillin. This choice was made at an expert meeting in Kathmandu in 2006, by a committee that included the Director General and representatives of the Department of Health Services, the president of the Nepal Paediatric Society, senior consultants from Patan Hospital, the Maternity Hospital, the Institute of Medicine, Kanti Children's Hospital, the Nepal Health Research Council and Save the Children US. For infants of less than two months of age with suspected serious bacterial infection, Amoxicillin is given orally in a dose of 62.5 mg twice daily for seven days. In cases where families agree to take their infants to health facilities, referral to health workers is followed by treatment with gentamicin at a dose of 5 mg/Kg intramuscularly, daily for seven days. The government health workers tasked with providing these intramuscular injections are already practised at administering immunisations. In order to reduce risk, they were trained specifically in gentamicin administration at the start of the intervention.

#### Supervision to ensure safety and to maintain quality

The interventions were devised by a team of paediatricians (DSM, AC and DO), the project manager (BS), who is trained to Health Assistant level, and a specialist in community mobilisation (NS). Whilst the interventions are conducted by community volunteers with no clinical training, safety of the sepsis intervention is ensured by involving existing government health workers in the supervision of the FCHVs and by making sure that FCHVs only refer infants to approved trained government health providers. In addition, each baby with suspected infection is visited by a MIRA supervisor who is trained to the level of either Auxiliary Nurse Midwife or Community Medical Assistant. In cases where parenteral antibiotics are required the supervisors encourage the family to take the baby to the health facility for intramuscular injections.

It could be argued that providing the FCHV with oral amoxicillin might delay the provision of parenteral antibiotics and therefore increase risk. The reality is that few families feel comfortable with taking newborn infants to health facilities, and with them receiving injections. This means that uptake of gentamicin treatment is low and that our intervention is unlikely to increase the risk over the community norm.

Regular reporting and meetings ensure that safety and adverse effects are monitored. Whilst no adverse effects of the women's group intervention were expected, preparation of health messages and materials (e.g. picture cards) was closely monitored to ensure compliance with current recommendations.

### Potential for contamination

There are two main sources of contamination, the first between interventions under test, and the second between intervention and control clusters.

#### Contamination between interventions

both the women's group intervention and the sepsis management intervention are being implemented through FCHVs. FCHVs who are trained to facilitate women's groups may develop better communication skills and become more mobilised to conduct their sepsis management activities than FCHVs who do not take part in the women's group intervention. Similarly, women who are involved in the groups may become aware of the sepsis management intervention and spread positive information in the community, which could make families more receptive to the FCHV's assistance when she visits on sepsis management work. On the other hand, the extra workload that women's groups impose on FCHVs could decrease the time they have to devote to making visits to identify, treat and follow-up sick newborns; or sepsis management activities could decrease the time available to undertake women's group activities. The extent to which women's groups enhance or detract from the implementation and impact of the sepsis management intervention (and vice versa) will be addressed by comparison of descriptive statistics for clusters implementing both interventions with those implementing single interventions and control clusters. We shall compare respondents who participated in women's groups with those who did not, and respondents who received sepsis treatment when their child was ill with respondents whose children fell ill but did not receive the intervention. Both process indicators (coverage of sepsis and women's groups) and outcomes will be examined in a process evaluation. Focus groups and semi-structured interviews with FCHVs, MIRA staff and recipients of the interventions will explore perceptions of how the interventions supported and interacted with one another in practice.

#### Contamination between intervention and control clusters

The likelihood of the sepsis management intervention contaminating non-sepsis intervention areas is low because each FCHV only works in her own ward. However, since the women's group intervention is based around a concept of messages being transferred within communities, there is potential for women's group messages to spread from intervention into control and non-study areas. This is increased because some women move between their marital and parental homes during the childbearing period (mid-pregnancy to around two months postpartum), which means they may move in and out of intervention areas and also in and out of study areas. This is something that we cannot control. We decided it was not feasible to create buffer zones between intervention and control areas to control contamination because the plains of Nepal are densely populated and relatively accessible, which means that messages may move quite long distances. In addition, after excluding small and politically disrupted clusters there were insufficient to choose from to evenly create buffer zones around intervention clusters. Selection of individual communities within VDC clusters would have decreased sample size and might have caused conflict in an already conflict-affected area.

As an alternative to creating buffer zones, the surveillance questionnaire was designed to detect contamination between interventions by asking respondents whether they had heard of or attended the women's groups and whether they had received sepsis management visits from FCHVs. The locations and amount of time spent in marital and parental homes during pregnancy and postpartum, and the location of the birth, were recorded so that we could estimate the exposure of each mother-baby pair to the interventions under test.

### Study duration

Trial activities cover a span of six years. Two years to mid-April 2007 were occupied with staff recruitment, planning, community entry and collection of baseline data. Baseline surveillance of vital events, births and deaths, using the same tools as those used during the trial, ran from 1 September 2006 to 13 April 2007 (the end of the Nepalese calendar year). Due to unexpected insurgency during 2007-8, interventions were allowed a run-in period of one year from 14 April 2007 to 12 April 2008 as they became established in the midst of considerable disruption. The full-scale intervention is running 13 April 2008 to 13 April 2011 (coinciding with Nepali calendar years 2064-2067). May - December 2011 will be taken up with an endpoint nutritional anthropometric study (not included in this protocol), sustainability activities, data cleaning, analysis and preparation for dissemination and publication.

### Randomisation and allocation

Originally, a sample size calculation was undertaken which indicated that 48 clusters should be sufficient for analysis of the primary neonatal mortality outcome. However, since the Maoist insurgency in Nepal was still very disruptive to field activities at the time of setting up the trial, we decided to randomise 60 clusters instead of 48 in case of loss to follow-up due to insurgency. This amounted to an extra 3 clusters in each of the allocation groups of women's group only, sepsis only, women's group and sepsis, and no intervention. 60 rural clusters were selected randomly from the 101 in the district (Janakpur municipality has been excluded from the study). Because of the ongoing conflict, eight village development committee areas were excluded from sampling on the basis of serious working difficulties. A further 14 were excluded because their populations were less than 4000. 60 clusters were then selected for the study - from a potential list of 79 - using a random number sequence generated in Microsoft Excel.

The first allocation (to the women's group intervention) was done in two strata. A stratum of eight very large village development committees was block randomized to either intervention or control, and the remaining stratum of 52 was treated similarly. In the second allocation, the two groups of 30 were block randomized to either sepsis management intervention or control.

### Measures

#### Primary outcome

neonatal mortality rates.

#### Secondary outcomes

##### MIRA Dhanusha community groups

stillbirth, infant and under-two mortality rates; care practices and health care seeking behaviour; maternal eating behaviour in pregnancy and postpartum; exclusive breastfeeding and complementary feeding; maternal and young child (under-2) anthropometric status (body mass index, Z scores for height-for-age, weight-for-age and weight-for-height).

##### MIRA Dhanusha sepsis management

identification and treatment of neonatal sepsis by community health volunteers, infection-specific neonatal mortality assessed by community verbal autopsy.

### Sample size

Sample size is based on the primary outcome of neonatal mortality rate. Calculations are based on the equations of Hayes and Bennett,[28] assuming two treatment groups, unmatched clusters of approximately equal size, a value of *k *- the between-cluster coefficient of variation - equal in intervention and control groups, and the addition of 2 to the estimated cluster number to account for loss of degrees of freedom consequent on stratification. The value of *k *was set at 0.3 on the basis of estimates from the MIRA Makwanpur study. A sample size of 48 clusters (24 intervention, 24 control), with an average 190 annual births documented over 2 years (i.e. 380 per cluster), would have 80% power to detect a 30% fall in neonatal mortality rate from 36.0 to 25.2 per 1000 at 95% significance level.

### Data collection

Data is collected from five sources, using pretested formats, modifications of which were adapted on the basis of field experience during the baseline data collection period of Sept 2006 - April 2007.

#### Source 1

District Development Committee and District Public Health Office records. These sources provided baseline data mainly based on projections from the 2001 Nepal census at the cluster level on demographics, socioeconomic status, livelihoods and health activities.

#### Source 2

MIRA Dhanusha vital registration system. We set up a surveillance system for all births and newborn, infant, child and female deaths in the 60 village development committee clusters covered by the study. The design and implementation of the surveillance system used in the MIRA Makwanpur study have been described and are available in programme publications [29]. We have implemented similar systems in rural Bangladesh[30], rural and urban India[18,31], and rural Malawi [32]. Field-based enumerators, recruited locally in cluster sub-areas, identify births and deaths, and interviewers who visit the household verify these vital events.

Individual interviews were collected for all 6292 births in the baseline period. Subsequently, 10 births identified in each month are selected randomly for interview. Interviewers usually visit at about four to six weeks after birth to administer the surveillance questionnaire and (except for periods where insurgency disrupts activities severely) questionnaires are usually taken before 3 months from the birth. The questionnaire collects information on socioeconomic factors including food security, demography, maternity history, antenatal, delivery and postnatal care, newborn care and health care seeking, maternal eating practices during pregnancy and postpartum, infant feeding practices and awareness of and exposure to the interventions under test.

For all maternal or under-5 child deaths, interviewers administer a verbal autopsy questionnaire together with selected sections of the birth surveillance questionnaire that do not overlap with information in the verbal autopsy. By the end of the trial we will have more than 2500 perinatal verbal autopsies, around 600 under-5 verbal autopsies, more than 100 maternal verbal autopsies and basic information on more than 400 non-maternal deaths of reproductive age women. In general a longer gap is allowed after deaths before verbal autopsy interviews are taken to allow the bereaved family time to mourn. Unless insecurity disrupts activities or the bereaved family refuses to discuss the death, verbal autopsies are usually completed within 3 months of the event. However in a small proportion of cases interviews may be taken as long as a year after the event once the family feels able to talk about it.

Analyses may account for sampling bias introduced by sampling all deaths and only a sample of births, by using weights based on the total numbers of births and deaths each month in each cluster, available from the vital surveillance dataset. If needed we may also control for recall bias by including a term for time after the event that questionnaires were taken.

The surveillance system was run for six months before the start of the interventions in order to develop a baseline picture (for infants born between 1 Sep 2006 and 14 April 2007) generating a total of c.6200 surveillance questionnaires. Around 6200 births or neonatal deaths are surveyed annually for each year of the trial making c.25 000 between the trial run-in year and the end of year 3 of the trial (i.e. between baby dates of birth or death 13 April 2007 and 13 April 2011).

The vital events database, which contains all vital events, will have c.6200 live and still births during the baseline period and c.11500 births per year during each year of the trial, making a total of c.46 000 live and still births to include in cluster level analyses over the 4 trial years (i.e. run-in year plus years 1 to 3).

#### Source 3

FCHV reporting system. Based on the experience of the MINI project, we strengthen and adapt the reporting system for FCHVs and health workers in the clusters in which the sepsis management intervention is introduced. The data include numbers of live births, stillbirths and deaths within two months that the FCHVs detect, and the proportion of infants who get the minimum requirement of 4 follow-up visits and birth weight measurement within 24 hours of delivery. In addition, numbers of low birth weight infants, details of danger signs identified, numbers of infants diagnosed with possible serious and possible local bacterial infections, numbers complying with amoxicillin treatment, number referred for gentamicin injection and numbers who received injections are recorded by MIRA health supervisors by scrutinising FCHVs' pictorial recording sheets. These data can then be compared with (i) vital surveillance data to estimate proportions of births and deaths identified in each FCHV's working area, and (ii) with caregiver's recall of FCHV activities in the sepsis management intervention areas.

#### Source 4

Ancillary studies and data collection. We collect information on household economy and dietary practices with targeted studies. Both quantitative and qualitative methods are used, depending on particular questions.

#### Source 5

Process evaluation data collection. We collect information on the context of the interventions, how they were implemented in theory and practice, the impact on group members, non-group members and communities. Detailed data on monthly group attendance, meeting summaries, problems prioritised, strategies being implemented and progress with strategy implementation are collected. Both quantitative and qualitative methods are used.

### Quality control

Vital events identified by enumerators are confirmed by interviewer visits to the household, first to verify the event and later to fill in a questionnaire. Field supervisors observe 10% of contacts. A data auditor checks forms for completeness and quality. Data are entered into an electronic relational database management system in Microsoft Access, and backed-up regularly on a server partition and on compact discs.

### Interim analyses

A Data Monitoring Committee (DMC) will meet in early 2011, according to DAMOCLES guidelines [33]. Unfortunately, restrictions of movement due to security issues during the 2007-2009 insurgency resulted in problems with database systems that could not be resolved early enough to hold a DMC at the preferred trial midpoint (early 2009).

#### Safety issues

We do not expect any negative effects of the women's group intervention. Harmful effects of the sepsis intervention have been minimised by (i) limiting the work of the FCHV to basic weight categorisation and diagnosis, (ii) FCHVs administering well-tested drugs in small doses, and (iii) by involvement of clinicians in supervising the intervention. Injections are only administered by trained health personnel within the existing health system, using individuals who would already be providing them routinely. Adverse events associated with FCHV management of neonatal illness are recorded.

### Analysis strategies

The analysis will be undertaken as intention-to-treat at both cluster and participant levels. Participants who begin the trial as residents of a given cluster will be retained as such even if they move to another cluster during the trial period. The analysis will describe participant flow, deviations from protocol, recruitment and potential sources of bias. A table will summarize baseline characteristics of control and intervention clusters.

Seven clusters were included in the original randomisation but excluded soon after because of political insecurity. No intervention activities took place in these clusters. As a secondary analysis we shall also analyse the trial findings with these clusters excluded from the analysis.

We will have two sources of data for the primary mortality outcomes, one nested within the other. First, births and deaths identified and summarized as cluster-level totals. Second, a sample of families (550 per month) visited to collect detailed interview information. Although this provides information on demography, socioeconomic status, care practices and outcomes, our presumption is that it will not exactly reflect population mortality figures, since there are systematic differences between the likelihoods of interviewing a bereaved family and a family whose infant has survived. For this reason, we suspect that that cluster-level figures are likely to give the most accurate estimates of mortality rates. We will therefore conduct two types of analysis: at cluster level and at individual level.

The cluster-level analysis will begin with two-sample t-tests, which are relatively robust to deviations from normality. We will present 95% confidence intervals for mortality rate differences. For event rates, we will fit a Poisson regression model that ignores the intervention effect, compare predicted with observed mortality rates, and examine the effect of the intervention through a t-test on the residuals[34]. For individual-level analysis, we will use random effects logistic regression grouped on clusters. If quadrature checks do not support the use of this approach, we will consider using Generalized Estimating Equations. Analyses will be adjusted for socioeconomic status and religion.

The factorial nature of the evaluation design assumes independence of effect of the two interventions on the primary and secondary outcomes. We do not think that the design will allow us to explore interaction beyond the speculative, and will present the findings in a table that compares the four combinations of intervention and control. Care practices, health care seeking behaviour, identification, treatment and referral of neonatal sepsis by FCHVs will be presented in tables of frequencies and percentages.

We will not know whether we have sufficient power to compare separately the impact on mortality outcomes of the first and second action cycles of the women's group until we see the effect size. We hope to analyse 2 blocks of 2 years (run-in year and year 1, and years 2-3) to see the effect of the first cycle and then that of the second in addition to the first. It will not be possible to analyse the mortality impact of the second cycle independently from that of the first, since strategies begun in the first cycle will continue during the second. We aim to compare the effect of the second cycle on nutrition-related behaviours in comparison with the effect of the first.

Qualitative information on activities resulting from women's groups will be analysed thematically. The trial findings will be presented in accordance with CONSORT guidelines[35,36].

### Ethical issues

#### Approvals

The study is being undertaken under a Memorandum of Understanding with the Government of Nepal Ministry of Health. It has ethical clearance from the Nepal Health Research Council and the ethics committee of the Institute of Child Health and Great Ormond Street Hospital for Children, UK.

#### Consent

After community-level meetings in each cluster, pre-allocation signed consent was taken from cluster guardians in the person of village development committee secretaries. Participation in either a community group or in the work of the FCHV is at the discretion of families in Dhanusha district. Participation in the study involves verbal consent to be interviewed about pregnancy, delivery and the newborn period. Participants give verbal consent and are free to decline to be interviewed at any time. All community-based members of the study team were recruited locally and carry out their activities in their home areas. When surveillance team members note minor illness in mothers or infants, they encourage attendance at an appropriate health facility. In the event of severe illness, team members have an ethical responsibility to assist with rapid and appropriate transport and treatment, regardless of allocation. All information provided by participants remains confidential. Access to information is limited to interviewers, supervisors, data auditors and officers, and research staff at the analytical level. No analyses or outputs will include the names of participants.

#### Benefits to control areas

In order to provide support to existing health services in both control and intervention areas, essential newborn care training to all health cadres, an equipment audit and provision of certain equipment for essential newborn care was provided to health facilities throughout Dhanusha district. Table 1 summarises the training and equipment provided to control and intervention groups to strengthen government health service provision for mothers and newborns.

**Table 1:** Training and equipment provided to control groups to strengthen government health service provision to mothers and newborns

*Audit*
All government health facilities
Newborn care equipment availability and function
*Equipment*
All government health facilities
Suction bulb
Weighing scale
Thermometer
Ambubag
All primary health centres
Warm cot
Resuscitaire
Phototherapy unit
Zonal hospital
2 warm cots
2 resuscitaires
2 phototherapy units
*Consumables*
All maternal and child health workers
2 wiping/wrapping cloths
Rubber suction bulb
Silicon tube and mask
Glass thermometer
30 ml betadine solution
30 ml gentian violet solution
Pictorial manual
*Training*
All government health workers in the district: essential newborn care

**Benefits in sepsis management intervention clusters only**
*Consumables*
All government health facilities in sepsis management intervention clusters:
Injection gentamicin 80 mg (10 vials)
Disposable syringe 1 ml (50)
Tab amoxicillin DT 125 mg (50 tabs)
Recording forms
*Training*
All government health workers in sepsis management intervention clusters: community-based sepsis management
All Female community health volunteers in sepsis management intervention clusters: essential newborn care and community-based sepsis management

#### Sustainability and scalability

Both interventions have been designed and modified with an emphasis on potential sustainability. MIRA trained all health personnel in the district public health system in essential newborn care and all health personnel in sepsis intervention areas in the management of newborn sepsis. National bodies have ratified the training modules and MIRA is the primary trainer for newborn care in Nepal. The Nepal Government will run subsequent training.

The involvement of FCHVs in both interventions represents a route to potential scale-up. Our experience in the MIRA Makwanpur trial was that it would be preferable for interventions to be conceived from the beginning in terms of best fit with existing human resources. FCHVs are already responsible for ward-level mothers' groups, and the MIRA Dhanusha community groups' intervention builds directly on this. In the MIRA Dhanusha sepsis management intervention, initial training, orientation and logistics are managed by MIRA, but in partnership with regular district health system activities. Monitoring and supervision will be transferred gradually to the district health system so that at the end of the study FCHVs will source drugs and supervision from local health institutions under the supervision of the district health office.

#### Public engagement

There will be a substantial increase in our knowledge about perinatal and neonatal events and nutrition, and an indication of whether demand-side interventions are effective, scalable and replicable. We hope that this research will help policymakers in Nepal and in other developing countries to make informed choices about the use of community health volunteers for treatment and referral of sepsis, and the use of a new cadre of reproductive health community health mobilisers to concentrate on the demand rather than the supply side obstacles to better maternal and child health. We also think that the findings of the study will have implications for the community component of the Integrated Management of Childhood Illness strategy and safer motherhood and newborn care programmes.

Additionally, the whole notion of the women's group intervention is predicated on potential sustainability and cost-effectiveness. All MIRA-CIHD collaborative research is carried out in partnership with the Government of Nepal Ministry of Health. Representatives of the Ministry (particularly the Family Health Division) and the Nepal Health Research Council have been involved in the development of our proposals to test expansion of women's group and community health volunteer activities. The MIRA-CIHD collaboration and the Ministry of Health meet regularly to discuss ongoing research and how it might influence national policy and practice. The trial findings will be published in an open access peer-reviewed journal.

### Project management and research governance

The MIRA-CIHD collaboration has a track record of over 18 years of successful partnership. Local MIRA employees control the management of research studies, and UK inputs are purely technical and advisory. MIRA handles all recruitment, administration and implementation aspects of the research. Established management tools and performance controls are used, including recruitment protocols, conditions of service and terms of reference for all cadres. Senior UCL CIHD team members visit MIRA for project review every three to six months. Scientific papers submitted for publication as a result of the studies will be subjected to peer review. The responsibilities of the Data Monitoring Committee will be to assess efficacy, adequacy of sample size, and comparability of treatment arms, and to approve the final analytic plan, including analysis that takes account of loss of clusters.

## Competing interests

The authors declare that they have no competing interests.

## Authors' contributions

All authors contributed information to drafts, read and approved the final manuscript, participated in the design of the study, and will participate in the analysis and interpretation of data. BPS is the project manager and is responsible for the management of the trial. BB is the senior officer responsible for the women's group intervention. NS is the UCL CIHD technical advisor to the study, prepared the final draft and gave final approval of the version to be published. DSM is the director of MIRA and AC of UCL CIHD. DO provides regular technical support to the Nepal-based team and wrote the first draft of the protocol.

## Abbreviations used in the text

MIRA: Mother and Infant Research Activities; FCHV: Female Community Health Volunteer; SEARCH: Society for Education, Action and Research in Community Health; WHO: World Health Organisation; MINI: Morang Innovative Neonatal Intervention; VDC: Village Development Committee; HIV/AIDs: Human Immunodeficiency Virus/Acquired Immunodeficiency Syndrome; DMC: Data Monitoring Committee; DAMOCLES: Data Monitoring Committees: Lessons, Ethics, Statistics, CIHD UCL Centre for International Health and Development, Institute of Child Health, London; UCL: University College London; DFID: UK Department for International Development; USAID: United States Agency for International Development
